# Investigation of Deep Spiking Neural Networks Utilizing Gated Schottky Diode as Synaptic Devices

**DOI:** 10.3390/mi13111800

**Published:** 2022-10-22

**Authors:** Sung-Tae Lee, Jong-Ho Bae

**Affiliations:** 1 Department of Electronic Engineering, Gachon University, Seongnam-si 13120, Gyeonggi-do, Korea; 2 School of Electrical Engineering, Kookmin University, Seongbuk-gu, Seoul 02707, Korea

**Keywords:** neuromorphic device, in-memory computing, hardware-based neural networks, deep learning, spiking neural networks, off-chip learning, gated Schottky diode, synaptic device

## Abstract

Deep learning produces a remarkable performance in various applications such as image classification and speech recognition. However, state-of-the-art deep neural networks require a large number of weights and enormous computation power, which results in a bottleneck of efficiency for edge-device applications. To resolve these problems, deep spiking neural networks (DSNNs) have been proposed, given the specialized synapse and neuron hardware. In this work, the hardware neuromorphic system of DSNNs with gated Schottky diodes was investigated. Gated Schottky diodes have a near-linear conductance response, which can easily implement quantized weights in synaptic devices. Based on modeling of synaptic devices, two-layer fully connected neural networks are trained by off-chip learning. The adaptation of a neuron’s threshold is proposed to reduce the accuracy degradation caused by the conversion from analog neural networks (ANNs) to event-driven DSNNs. Using left-justified rate coding as an input encoding method enables low-latency classification. The effect of device variation and noisy images to the classification accuracy is investigated. The time-to-first-spike (TTFS) scheme can significantly reduce power consumption by reducing the number of firing spikes compared to a max-firing scheme.

## 1. Introduction

Deep learning has shown astonishing achievements in a broad range of tasks [1,2,3,4,5], such as image classification and speech recognition. However, recent high-performance deep neural networks require the high-performance computing resources like GPUs [6,7,8,9,10,11]. The enormous computational requirements limit the application of deep learning in an edge device. The spiking neural network (SNN) is a promising candidate for low-power hardware by mimicking neural network architectures of a human. Recently, deep spiking neural networks (DSNNs) have been actively studied to implement biologically plausible neural networks and large-scale neuromorphic computing platforms based on specialized analog hardware [12,13,14,15,16,17,18,19,20]. Hardware-based neural network platforms are much more efficient than CPU and GPU in terms of power consumption for running DSNNs and allow asynchronous and distributed event-driven computation, thereby improving scalability and reducing latencies. Furthermore, event-based asynchronous hardware-based neural network systems utilize their computational power on local active parts of the neural networks, and they efficiently save power of the entire neural networks. Therefore, a DSNNs-based neuromorphic system utilizing synapse and neuron devices is a promising candidate as a platform to run large-scale neural networks in real-time applications.

SNNs can be trained by spike-based on-chip learning algorithms, such as back-propagation and spike-timing-dependent plasticity (STDP) [21]. On the other hand, in DSNNs, off-chip learning is used with conventional ANNs followed by a conversion in which SNNs can be converted from conventional ANNs, which can utilize well-developed training methods and fully trained models of ANNs [22,23,24,25,26]. DSNNs have been a driving factor in the development of many modern computer vision and mobile robots with continuous high-dimensional observation and action spaces [24]. A previous work [22] analyzed the reason of accuracy degradation in detail and proposed weight normalization method to prevent data loss and achieved high inference accuracy close to the inference accuracy of software-based ANNs.

In this work, we investigate the hardware neuromorphic system of DSNNs based on the gated Schottky diode. Note that 2-layer fully connected neural networks are trained for the Modified National Institute of Standards and Technology database (MNIST) image recognition using gradient descent. The trained weights are imported into the hardware system modeled by the characteristics of the fabricated gated Schottky diode used as a synapse device [27,28]. The effective Schottky barrier height of the gated Schottky diode is modulated by the bottom gate bias or program pulses. The Schottky reverse current is exponentially proportional to the amount of stored charge, and the amount of stored charge is logarithmically proportional to the number of applied pulses. These exponential and logarithmic relationships cancel each other out, and this can provide the excellent linearity between the reverse current and the number of applied pulses. There is a trade-off between the endurance and retention characteristics of the devices, which needs to be considered in device design [29,30]. Near-linear conductance response of the gated Schottky diode makes it easy to transfer quantized weights from software to hardware synaptic devices. We propose an adaptation of the neuron’s threshold to reduce the accuracy degradation due to the conversion from ANNs to DSNNs. In addition, we show that the left-justified rate coding method can considerably reduce the number of firing spikes compared to the right-justified rate coding, while achieving the same inference accuracy. The effect of the weight variation and noise in the input images on the classification accuracy is investigated. Finally, we show that the time-to-first-spike (TTFS) scheme can considerably reduce power consumption compared to the max-firing scheme by decreasing the number of spikes.

## 2. Materials and Methods

**Figure 1:**
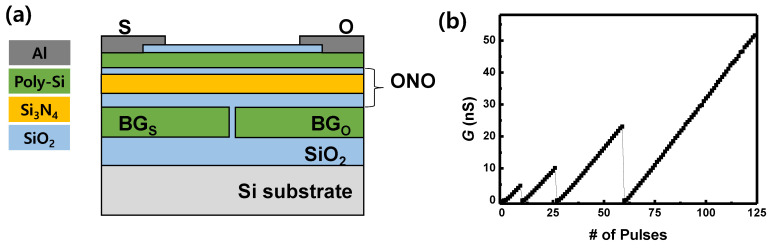
(**a**) A cross-sectional schematic diagram of the fabricated GSD. (**b**) Conductance response of GSD with the number of pulses when *V*_PGM_ (9 V, 10 μs) is applied to BG_S_ for 10, 15, 35, 64 times in sequence.

The gated Schottky diode (GSD), which is used as synaptic devices, is fabricated [27]. Figure 1a shows a schematic diagram of a fabricated GSD. The minimal unit cell size is 6F^2^ for one GSD device and 12F^2^ for one synaptic device. Two electronic devices are required to represent a unit synapse, because the weights of the unit synapse in neural networks should have positive and negative values for high accuracy. O and S represent the metal electrodes for ohmic-like junction and Schottky junction, respectively. BG_O_ and BG_S_ represent the bottom gates under O and S, respectively. The main device fabrication steps are described below, and the detailed fabrication steps are described in the previous work [14]. On top of a 300 nm thick buried SiO_2_, an *n*^+^ poly silicon layer and Si_3_N_4_ layer, which are a bottom gate and a sacrificial layer, respectively, were deposited and patterned. A SiO_2_ layer was thermally grown along the sidewall of the patterned *n*^+^ poly silicon to isolate the bottom gates. After that, another *n*^+^ poly-Si layer was deposited, followed by chemical mechanical polishing and patterning *n*^+^ poly-Si to isolate the bottom gates. Then, the sacrificial Si_3_N_4_ was stripped by H_3_PO_4_ at 160 °C. A SiO_2_/Si_3_N_4_/SiO_2_ (12/6/6 nm) charge trap stack was deposited and a 20 nm thick undoped poly silicon active layer was formed. After passivating the device with SiO_2_, the contact holes for the S, O and bottom gates were formed and Al electrodes were deposited and patterned by thermal evaporation and a lift-off process.

By applying pulse to the bottom gate under the S electrode, the charge stored in the Si_3_N_4_ layer is changing, which modulates the Schottky barrier and the conductance of the GSD. As GSD operates as a reverse diode, the output current is represented by the reverse Schottky diode current (*I*_R_). Figure 1b represents the conductance (*G*) response of the GSD with respect to the number of applied pulses when *V*_PGM_ (9 V, 10 μs) is applied to BG_S_ 10, 15, 35, 64 times in sequence. The conductance response shows excellent repeatability and linearity. Electrons are stored in the Si_3_N_4_ layer when *V*_PGM_ are imposed, which decreases the Schottky barrier height and increases *I*_R_ and G, and vice versa. *I*_R_ is exponentially proportional to the Schottky barrier height (equivalently the amount of stored charge), and the amount of stored charge is logarithmically proportional to the number of applied pulses. These exponential and logarithmic relationships cancel each other out, and this can provide the excellent linearity between the *I*_R_ and the number of applied pulses [27].

## 3. Results

In DSNNs, off-chip learning is used with conventional ANNs followed by a conversion in which SNN is converted from conventional ANNs, which can utilize well-developed training methods and fully trained models of ANNs. A previous work [13] analyzed that the reason of accuracy degradation occurred in conversion from ANNs to SNNs in detail and proposed a weight normalization method to prevent data loss and achieved the result close to that of software-based ANNs. In addition, [31] applied a weight normalization method to hardware-based DSNNs. On the other hand, we propose rescaling of the neuron threshold and apply it to the hardware-based DSNNs, which improves the learning accuracy. As the number of neuron devices is much smaller than that of the synapse device, it is easier to adapt the threshold voltage (*V*_th_) of the neuron device than to adapt the conductance of the synaptic device. The split-gate positive feedback device can be used as a neuron device which can adapt the threshold of neuron [32].

**Figure 2:**
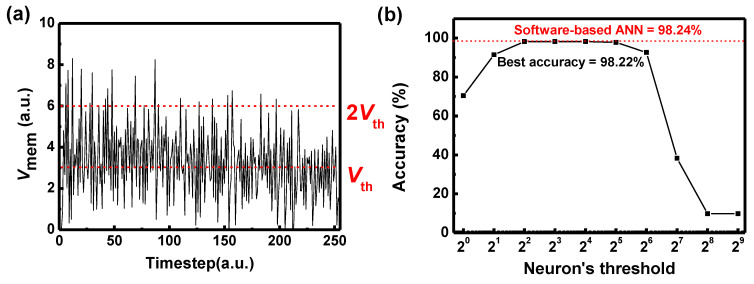
(**a**) The voltage of neuron membrane with respect to the timestep. (**b**) Inference accuracy with respect to the neuron’s threshold in DSNN.

The multilayer neural network of 784-200-10 is trained for the MNIST dataset using a backpropagation algorithm, achieving an inference accuracy of 98.24%. Then, the trained weights are transferred to hardware-based DSNNs of 784-200-10, reflecting conductance response of the GSD device in Figure 1b. The weights of synapse in the system have values from 1 to −1, and the magnitude of input spike is 1 or 0. Figure 2a shows the voltage of neuron membrane (*V*_mem_) with respect to the timestep as an example. As the MNIST dataset has a resolution of 8-bit, the input spike in DSNNs has 255 timesteps. As shown in Figure 2a, *V*_mem_ can exceed two times of the *V*_th_ of a neuron device. However, the neuron device cannot generate two spikes at one timestep, which results in data overflow and decreases the inference accuracy of neural networks. In this case, the threshold of neuron is low and should be increased to prevent overflow of data. On the other hand, when the *V*_th_ of a neuron device is too high, the *V*_mem_ of the output neuron cannot reach to the *V*_th_ until the last input spike is transferred to the output neuron. In this case, the neuron cannot fire, which results in data underflow and decreases the inference accuracy. The *V*_th_ of the neuron device should be decreased to prevent underflow of data. As shown in Figure 2b, the inference accuracy is low when the threshold of the neuron is too high or too low due to the data underflow and overflow, respectively. Inference accuracy of 98.22% is achieved by optimizing the *V*_th_ of the neuron device, which is similar to the inference accuracy of software-based ANN.

**Figure 3:**
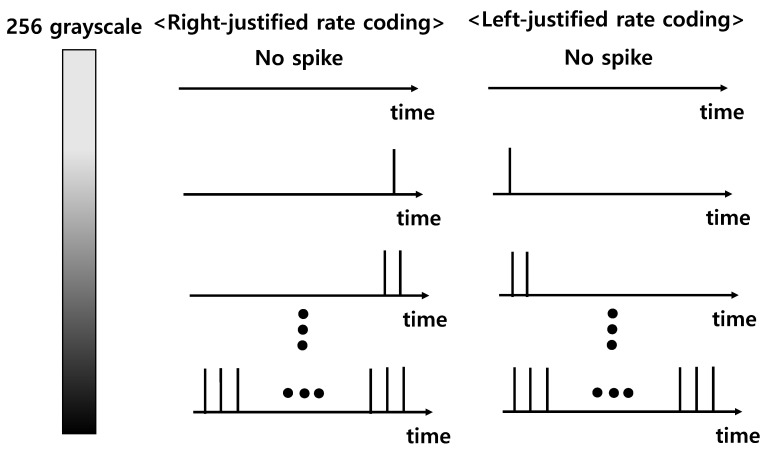
Schematic view of the RRC and LRC schemes. Grayscale is expressed by the number of spikes, and LRC and RRC indicate the cases where the spike is filled from the first time and the last time, respectively.

**Figure 4:**
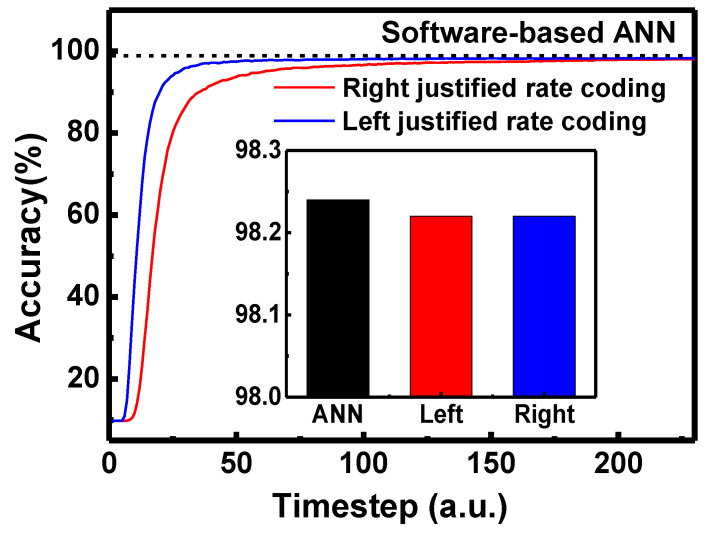
Comparison of inference accuracy in RRC and LRC. The inset shows the final inference accuracy of ANN, LRC and RRC.

To convert input value of software-based ANN to time-series spikes of DSNNs, there are two types of encoding methods, which are rate-based coding and time-based coding [33,34]. In addition, there are the max-firing scheme and TTFS scheme in rate-based coding [35,36]. In the max-firing scheme, the neuron which generates the maximum number of firing spikes in the last neuron layer is selected as a result. There are right-justified rate coding (RRC) and left-justified rate coding (LRC) in rate-based coding, as shown in Figure 3 [31]. As the MNIST image has 256 grayscales, the timesteps of rate coding in DSNNs are 255. The intensity of image pixel is proportional to the number of spikes. RRC fills the spikes from the last timestep, but LRC fills the spikes from the first timestep. In RRC, the last input spike should be generated at the last timestep. On the other hand, in LRC, the last input spike can be generated before the last timestep. Therefore, except for the grayscale of 256, the last spike in LRC is generated earlier than the last spike of RRC. Therefore, in LRC, input spikes generated from the pre-synaptic neuron can be reached to the post-synaptic neuron faster than in RRC. Figure 4 shows the inference accuracy of hardware neural networks when RRC and LRC are used. As shown in Figure 4, the LRC method can achieve high inference accuracy faster than the RRC method. In addition, the LRC method can achieve the same final accuracy as the RRC method.

**Figure 5:**
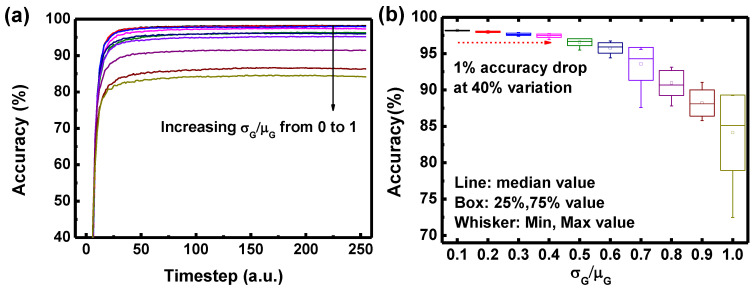
(**a**) Inference accuracy with increasing σ_G_/μ_G_ from 0 to 1. (**b**) Final inference accuracy as a function of device variation (σ_G_/μ_G_).

We estimate the effect of device variation (σ_G_/μ_G_) to the inference accuracy, as shown in Figure 5. The device variation which is assumed to follow Gaussian distribution is applied to hardware-based DSNNs. As shown in Figure 5a, in all the cases of σ_G_/μ_G_, the inference accuracy reaches saturated accuracy during the timestep, which means that DSNNs can perform stable operation even with σ_G_/μ_G_. On the other hand, saturated inference accuracy decreases as σ_G_/μ_G_ increases. In Figure 5b, simulation is repeated 20 times for each σ_G_/μ_G_. The median value of inference accuracy decreases as σ_G_/μ_G_ increases. The inference accuracy decreases by 1% when the σ_G_/μ_G_ increases to 0.4.

**Figure 6:**
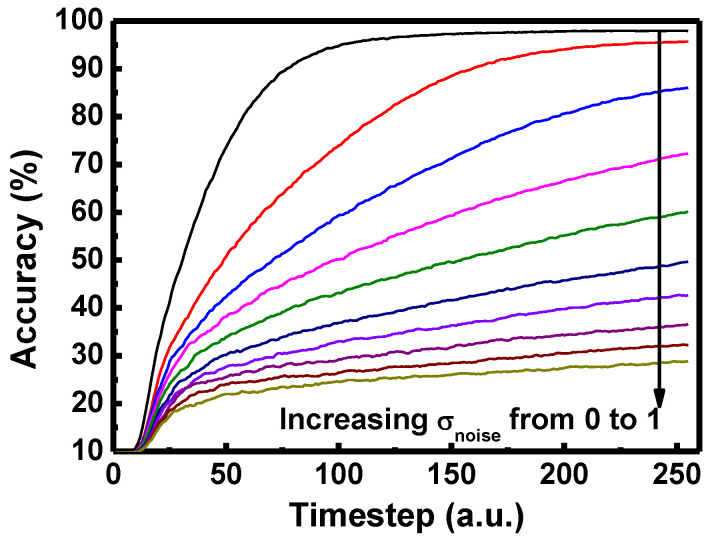
Inference accuracy over inference time of DSNN for various standard deviations of input noise (σ_noise_).

Input noise which follows Gaussian distribution with zero mean and standard deviation (σ_noise_) is added to the input image of MNIST dataset to investigate the effect of input noise to the inference accuracy. Figure 6 shows the inference accuracy of neural network with respect to various standard deviation of input noise. It is worth noticing that the inference accuracy does not saturate with the timestep as standard deviation of noise increases. On the other hand, the inference accuracy reaches saturated accuracy when the device variation exists. Therefore, the effect of input noise is more detrimental to the inference accuracy than the effect of device variation.

**Figure 7:**
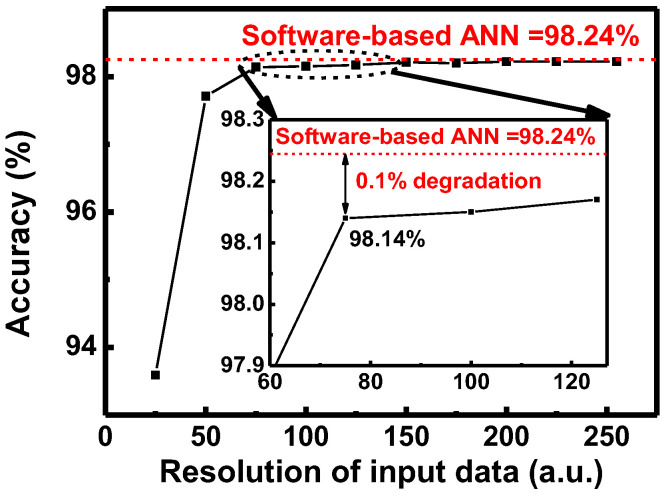
Inference accuracy with the resolution of input data. The inset is an enlarged plot to show the accuracy drop of the DSNN compared to the accuracy of the software-based ANN.

In addition, the effect of the number of input spike to the inference accuracy is investigated, as shown in Figure 7. The number of input spikes represents the resolution of input data. The MNIST dataset has 8-bit resolution, which corresponds to the 255 timesteps in DSNNs. As the number of timesteps decreases from 255, the data loss occurs in the conversion from MNIST image in ANN to the input spikes in DSNN. Therefore, inference accuracy decreases as the resolution of input data decreases from 255. The inference accuracy decreases by only 0.1% as resolution of input data decreases from 255 to 75. However, inference accuracy significantly decreases when the number of input spikes decreases from 75 to 50. Therefore, the resolution of input spikes should be greater than 75 steps to achieve sufficiently high inference accuracy.

**Figure 8:**
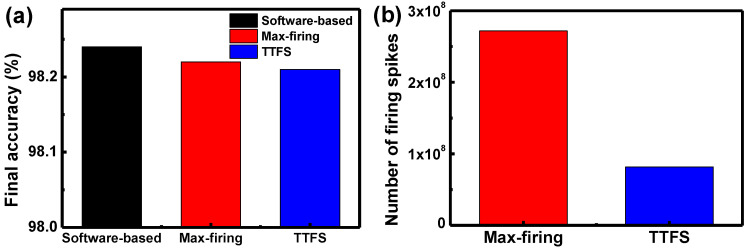
(**a**) Inference accuracy of software-based ANN and DSNN when the max-firing scheme and TTFS scheme are used. (**b**) The number of firing spikes when the max-firing scheme and TTFS scheme are used.

As described above, there are two types of schemes, which are the max-firing scheme and the time-to-first-spike (TTFS) scheme in rate-based coding. Up to now, we have investigated DSNNs based on the gated Schottky diode using the max-firing scheme. In the max-firing scheme, neuron which generates the maximum number of firing spikes in the last neuron layer is selected as a result. Therefore, the DSNN system needs counter circuits to add the number of firing spikes. In addition, the system counts the number of firing spikes until the last timestep in the last neuron layer. On the other hand, in the TTFS scheme, the neuron that generates the spike first is selected as a result, which does not need a counter. In addition, when the firing neuron first appears in the DSNN system, then the system does not enter further input data and the firing spike is not generated anymore. Therefore, it can significantly reduce the number of firing spikes and power consumption compared to the max-firing scheme. Figure 8a shows the inference accuracy of a software-based neural network and hardware-based neural network when the max-firing scheme and TTFS schemes are used. As shown in Figure 8a, the inference accuracy of 98.21% is achieved in the TTFS scheme used, which is comparable to the inference accuracy of the max-firing scheme. On the other hand, the TTFS scheme can considerably reduce the number of firing spikes than the max-firing scheme, as shown in Figure 8b.

## 4. Conclusions

In this work, we investigated the DSNN using the gated Schottky diode as synaptic devices. We proposed an adaptation of a neuron’s threshold to reduce the degradation of inference accuracy due to the conversion from ANNs to SNNs. The LRC method can achieve high inference accuracy faster than the RRC method, while achieving the same final accuracy to the RRC method. The effect of device variation and input noise to the DSNN system was investigated. The inference accuracy decreases only by 1% when the device variation increases to 0.4. The effect of input noise is more detrimental to the inference accuracy than the effect of device variation. In addition, the resolution of input spikes should be greater than 75 steps to achieve sufficiently high inference accuracy. Finally, the TTFS scheme can considerably reduce the number of firing spikes compared to the max-firing scheme, while achieving comparable inference accuracy to the max-firing scheme. The main limitation of this work is that the proposed scheme is verified by neural network simulation. The proposed scheme in this work can be validated by implementing a neuromorphic chip for deep spiking neural networks. In future work, the neuromorphic chip will be implemented utilizing synaptic devices and neuron devices where the proposed method in this work can be applied and verified.

## Data Availability

Not applicable.
